# Social singing, culture and health: interdisciplinary insights from the CHIME project for perinatal mental health in The Gambia

**DOI:** 10.1093/heapro/daab210

**Published:** 2022-02-16

**Authors:** Lauren Stewart, Bonnie B McConnell, Buba Darboe, Vivette Glover, Hajara B Huma, Katie Rose M Sanfilippo, Ian Cross, Hassoum Ceesay, Paul Ramchandani, Victoria Cornelius

**Affiliations:** 1 Psychology Department, Goldsmiths, University of London, London, UK; 2 School of Music, Australian National University, Canberra, ACT 2601, Australia; 3 The Ministry of Health and Social Welfare, Banjul, The Gambia; 4 Institute of Reproductive and Developmental Biology, Imperial College London, London SW7 2AZ, UK; 5 Centre for Music and Science, University of Cambridge, Cambridge CB3 9DP, UK; 6 The National Centre for Arts and Culture, Banjul, The Gambia; 7 PEDAL Research Centre, Faculty of Education, University of Cambridge, Cambridge CB2 8PQ, UK; 8 Imperial Clinical Trials Unit, School of Public Health, Imperial College London, London SW7 2AZ, UK

**Keywords:** interdisciplinary, health intervention, community, participatory music, singing, Kanyeleng

## Abstract

Arts in Health initiatives and interventions to support health have emerged from and been applied to mainly WEIRD (Western, Educated, Industrialized, Rich and Democratic) contexts. This overlooks the rich cultural traditions that exist across the globe, where community groups often make prolific use of participatory song and dance as a part of ceremonies, ritual and gatherings in everyday life. Here, we argue that these practices can provide a valuable starting point for the co-development of health interventions, illustrated by the CHIME project for perinatal mental health in The Gambia, which worked with local Kanyeleng groups (female fertility societies) to design and evaluate a brief intervention to support maternal mental health through social singing. Here, we use the project as a lens through which to highlight the value of co-creation, cultural embeddedness and partnership building in global health research.

## INTRODUCTION: BROADENING THE LENS OF ARTS IN HEALTH

Arts in health approaches are becoming increasingly recognized as powerful and versatile tools for health. The World Health Organization (WHO) has reviewed the global evidence linking the arts and health and well-being (Fancourt and Finn, 2019) and confirms that engaging with the arts has the potential to exert a positive influence on both mental and physical health across the lifespan. However, the frameworks used emerge mainly from WEIRD (western, educated, industrialized, rich and democratic) contexts, resulting in a skewed and narrow understanding of what constitutes the ‘arts’ (Roseman, 2011). Taking music as an example, for the world’s WEIRD population (estimated to comprise just 12% of the global population; Henrich *et al.*, 2010), engagement typically means attending concerts and/or curating their own online music collections, which they often listen to as a private experience. In contrast, the remainder of the global population also enjoys musical practices that tend to be more collectivist, participatory and deeply rooted in local culture and customs (Turino, 2008). They also take varied forms and fulfil diverse uses, though one consistent function apparent across many different sets of musical practices is that of equilibrating participants’ sense of self with social norms [see, e.g. (Blacking, 1967; Turino, 2008; Rudge, 2019)]. This does not negate the potential and power of the current conceptualization of arts in health but rather necessitates a reconsideration of what the arts are for the majority of the world’s population, in order to realize the enormous potential of existing local practices and traditions for health promotion in a way that is culturally embedded, meaningful and sustainable.

Approaches to health promotion through participatory arts and culture must also, by definition, take an interdisciplinary, partnership building perspective. Currently, such approaches are in the minority: in general, global health projects are typically led by medical practitioners, often trained in western contexts using frameworks and tools of the Western biomedical model, prioritizing quantitative measurement and efficacy and typically observatory rather than participatory. Approaches prioritizing more qualitative, narrative-based understandings are less frequently seen in low- and middle-income country (LMIC) contexts. The distinctly different outputs emerging from different disciplines remain largely siloed from one another—published in different journals and communicated to almost entirely different audiences (Magee and Stewart, 2015). From a global health perspective, a lack of interdisciplinarity is a missed opportunity, leading to less effective interventions (Rosenfield, 1992; Choi and Pak, 2006). Research has shown where there is failure to take into account local beliefs, attitudes and traditions, there is low uptake and/or acceptability of initiatives and interventions (Padmanathan and De Silva, 2013), translating into high costs in terms of human effort, economic investment and failure to deliver significant health improvements. Going further, we would argue that while a consideration of local context and culture is paramount for global health initiatives, approaches which explicitly take local context and culture as their *starting point* offer a great deal of promise. The utilization of cultural and community assets for health improvement has recently become a strategic priority within the UK (e.g. 2021 Integrated Care Services parliamentary white paper) but opportunities for this are arguably even greater in LMIC contexts, where both collectivist cultural practices as well as the lack of health infrastructure often presents fertile conditions for the organic emergence of approaches that utilize culture and community to support individuals emotionally, psychologically and even financially [e.g. (Barz and Cohen, 2011; Okigbo, 2016)]. In many cases, such practices often fly under the radar of the global health community because the traditional model has been to export and adapt approaches developed from WEIRD contexts rather than to take existing local practices as a starting point. The former approach can be limited by fundamentally different cultural frameworks, practices and belief systems which may render approaches developed outside of those cultures at best difficult to implement (feasibility and acceptability) as well as ineffective (Kola *et al.*, 2021). In contrast, we argue that using existing cultural and community-based practices as a starting point for the co-creation of theoretically driven interventions with measurable outcomes offers considerable promise, but critically depends upon achieving a deep level of understanding of those practices, and building partnerships with community and cultural organizations that are wide-ranging, equitable and sustainable (Rahman *et al.*, 2013).

Below, we describe a recent project which used existing local community and cultural practices—specifically coming together through participatory singing—as a starting point for the co-design of an intervention with measurable health outcomes. Funded by both the Medical Research Council and Arts and Humanities Research Council, the Community Health Intervention through Musical Engagement (CHIME) project (Sanfilippo *et al.*, 2020) was explicitly interdisciplinary, involving a wide range of project partners across geographical and disciplinary boundaries. Below, we discuss the clinical context of the project (alleviating the symptoms of anxiety and depression in pregnancy), the ethnomusicological starting point for the project (traditional musical practices of Kanyeleng fertility societies), the theoretical rationale for the intervention (mechanisms of group singing for mood regulation and social bonding), a brief overview of the method and the partnership building and co-creation work that resulted from the intervention. Since this work has been already published, only a brief summary is given here, as a context to discussing the wider issues of co-creation, cultural embeddedness and partnership building. We conclude with considerations and recommendations for future initiatives that wish to use culturally embedded participatory arts as a starting point for targeted health interventions.

## SUMMARY OF THE CHIME PROJECT

### Clinical context

High levels of prenatal anxiety and depression affect roughly 10–20% of women, which is comparable to the incidence of postpartum depression (Milgrom *et al.*, 2008). Maternal mental health problems can have adverse effects on foetal and infant development (Glover, 2015). High-stress levels during pregnancy also affect foetal and infant development (Glover *et al.,* 2009; Braithwaite *et al.,* 2015). Additionally, maternal stress and depression can have adverse effects on infant attachment security (Coyl *et al.*, 2002), with implications for infant developmental outcomes (Murray *et al.*, 1996). High levels of prenatal anxiety and depression are even more prevalent in LMIC contexts, most likely due to the exacerbating effects of poverty and stigma (Sawyer *et al.*, 2010) and a scarcity of mental health specialists means that there is an urgent need to develop effective, low-cost, non-stigmatizing and culturally appropriate interventions to support women’s mental health during the perinatal period. Our interest was to develop an intervention that was *universal*—rather than targeted at only women with high levels of symptoms. Universal interventions are in line with suggestions to move beyond a binary approach to mental health, towards a more dimensional one. This allows for such interventions to incorporate a ‘treatment’ of mental ill-health in those with high symptoms, along with the maintenance of good mental health and the prevention of future poor mental health in those who may have fewer symptoms, or none at all (Patel *et al.*, 2018).

### Ethnomusicological starting point

In The Gambia, traditional Kanyeleng groups consist of women who have experienced infertility or child mortality (Saho, 2012; McConnell, 2015, 2020). Kanyeleng groups make prolific use of participatory music and dance (as well as prayer and ritual) aimed at preventing infertility and infant mortality (Skramstad, 2008; McConnell, 2020). Although many Kanyeleng groups continue to practice traditional rituals, they have begun to assume prominent roles as health communicators, supported by the Ministry of Health (McConnell, 2020). In contemporary society within The Gambia, Kanyeleng groups perform in and beyond their local communities, disseminating information about HIV/AIDS (McConnell, 2015), vaccinations (McConnell and Darboe, 2017), diarrhoea, female genital cutting and breastfeeding (McConnell, 2016), which are relevant for pregnant women and new mothers. The Kanyeleng groups’ historical and continuing focus on women’s reproductive health, coupled with their expertise as musicians and health communicators, provides a potent example of traditional musical practices used in conjunction with contemporary medical knowledge to foster enhanced maternal and infant health. For these reasons, we selected four Kanyeleng groups as partners in co-development and delivery of the CHIME intervention.

### Theoretical rationale

Research from high-income contexts has shown that making music together can improve mood and help people feel socially close and supported (Williams *et al.*, 2018). Equally, music, specifically song, is an effective means of communication of messages, particularly in cultures where literacy rates are low, and performing messages is known to be an effective and engaging way to communicate important health messages (Panter-Brick *et al.*, 2006; Bastien, 2009; Bunn *et al.*, 2020). The use of social singing to improve mood, promote social bonds and convey information aligns well with recommendations from the WHO’s Mental Health gap action programme for LMIC contexts (Keynejad *et al.*, 2021) and formed the basis for the intervention.

### Methods

To inform the intervention development, we conducted ethnographic research, involving interviews and focus group discussions with pregnant women, midwives, community birth companions, Kanyelengs and griots (hereditary musicians). We also engaged in participant observation through participating in and observing naming ceremonies and Kanyeleng rituals, building on the longer-term research and knowledge of the partner organizations. Our aim was to develop an understanding, both of how mental distress during the perinatal period is understood and experienced in the Gambian context, as well as the existing musical practices associated with the perinatal period, and attitudes towards them. This allowed us to develop an understanding of the local language terms that are used to describe mental distress and mental wellbeing, which was important in avoiding stigma and encouraging engagement with the topic. The development of the intervention was informed by principles of co-design and participatory ethnography (Lavrencic *et al.*, 2021). The key feature was the bringing together of mental health expertise with the Kanyeleng groups’ expertise in participatory music-making. The co-design workshops involved knowledge sharing and intervention development based on the indigenous methodologies of Kanyeleng groups (i.e. singing, dancing, joking, and praising). In this way, the Kanyeleng groups were empowered to run the intervention in a participatory manner and to draw on existing repertoires aligned with the identified themes of the intervention program, namely the importance of social support as well as strategies to deal with some common physical and psychological challenges of pregnancy. Following intervention development, the trial was conducted in a stepped wedge design. Pregnant women, unselected for mental health status, were recruited from antenatal clinics and assigned to the intervention or control arms of the trial. Those in the intervention arm attended 6 weekly sessions at their local antenatal clinic, led by a Kanyeleng group local to them who had taken part in the co-development workshops. Local research assistants collected depression and anxiety scores before and after the intervention (or equivalent time period for the control participants) using the Self Reporting Questionnaire (Beusenberg and Orley, 1994), a WHO developed instrument for global use which uses a simple binary response scale to index symptoms of depression, anxiety and psychosomatic complaints. In addition to the quantitative data collected, 36 participants were interviewed to achieve a fuller understanding and evaluation of the intervention and thematic analysis was used to extract themes (Braun and Clarke, 2006).

### Results

**Fig. 1:**
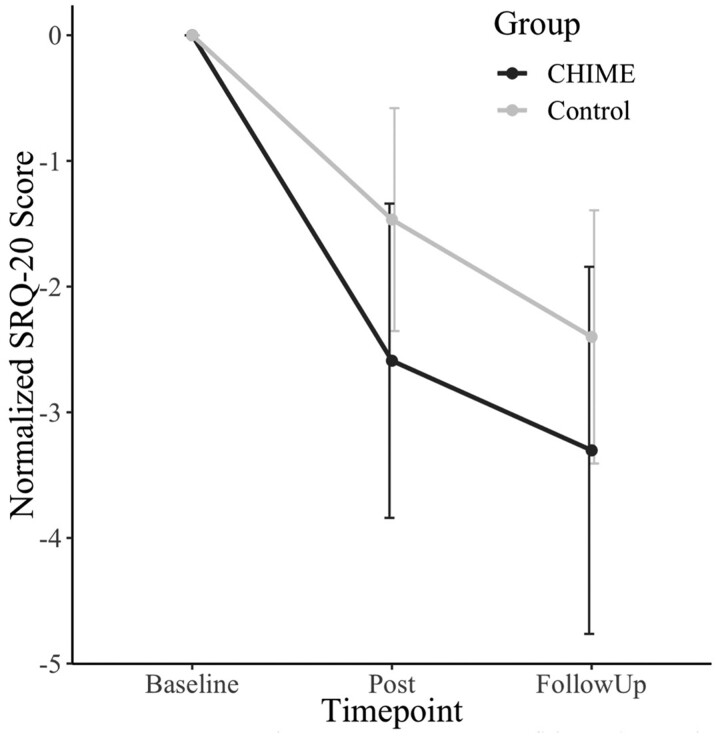
The effects of participatory music-based intervention for antenatal women in The Gambia at baseline (mid gestation), post (after 6 weeks of intervention) and at follow-up (4 weeks after the end of the intervention). Self-Reporting Questionnaire (SRQ)-20 scores for symptoms of common mental disorders were reduced more in the intervention (CHIME) group (*n *=* *39) versus the Control group (*n *=* *60) at post-intervention (*p *<* *0.01). SRQ-20 scores also reduced more at follow-up in the intervention (CHIME) group (*n *=* *33) compared with the Control group (*n *=* *50) (*p *<* *0.01). Error bars represent 95% intervals.

A significant reduction of symptoms was found, in comparison with a control group of pregnant women who did not receive the intervention (see Figure 1). In addition, the qualitative analysis revealed five higher level themes: Social Relationships, Peaceful Mind, Learning, Evaluations and Suggestions for the Future (see e.g. Table 1). For full details, see the original research article (Sanfilippo *et al.*, 2020).

**Table 1:** Themes and examples extracted from thematic analysis of interviews with intervention participants

Higher level theme	Category	Codes	Description: when a participant discusses…
Learning			… learning something new
	Care for baby		… learning information, which will help once the child is born
	Coping		… learning different ways of coping or seeking social support
	Health Information		… learning new health information, either about physical health or mental health
	Music		… learning new music
Peaceful Mind			… the positive impact the intervention had on a participants’ state of mind by giving her a peaceful mind or feelings of happiness
	Continue outside session		… the effect of the group continuing after the session is over
Social Relationships			… how the sessions have impacted social relationships
	Outside Relationships		… how the sessions impacted relationships with people outside the group itself
		Husband	… the intervention having an impact on the relationship with the husband
		Research Team	… the intervention having an impact on the relationship with the research team
		Teaching others	… the intervention having an impact on the relationship with those in the community by teaching and helping others
	Part of the singing group		… feeling a part of the intervention group
Suggestion for Future			… a suggestion for the future
	Attendance and Participation		… a suggestion that would help with attendance or participation
		Payment	… a suggestion about the payment for the pregnant participants or the Kanyeleng
		Transportation	… a suggestion about transportation
	Breakfast		… a suggestion about offering food as part of the intervention
	Continuation		… a suggestion to continue the intervention or the length of the intervention
	Timing		… a suggestion about when the sessions start and how long they last
Evaluation			… and evaluates the intervention
	Negative/Neutral Evaluation		… a negative or neutral evaluation of the intervention
	Positive Evaluation		… a positive evaluation of the intervention
		Music and performance	… the music or the Kanyeleng women in a positive way
		Session Structure	… a positive evaluation of the session structure and length

## DISCUSSION

### Partnership building across disciplines

The project spanned a number of academic disciplines, from ethnomusicology to psychiatry, cognitive psychology and medical statistics. Non-academic local partners were represented by diverse organizations including the Ministry of Health and the National Centre for Arts and Culture. The involvement of this broad range of disciplines and partner organizations was critical in a number of concrete ways: for instance, the National Centre of Arts and Culture gave us access to unparalleled linguistic expertise which was crucial for translating and back-translating our questionnaires and measurement tools into the two local languages. The involvement of the Ministry of Health and Social Welfare allowed us unprecedented access to antenatal clinics as well as to community workers who did on the groundwork to ensure that communities, including village elders (typically the leaders of the community), were aware of the project and had given it their support. In terms of research design, it was important to marry both clinical trial considerations (for instance, the timing of data collection in a ‘stepped-wedge’ design) with the logistical constraints of cultural and geographical considerations. The holy month of Ramadan (when musical participation is discouraged) is observed by the largely Muslim population, whereas the rainy season also has implications for participants to travel and or gather. A nuanced understanding of local customs and culture, along with knowledge of clinical trial design principles was vital for determining the final timeline for data collection that was respectful of local culture, feasible and rigorous.

### Recommendations for future culturally embedded participatory arts for health initiatives

In many non-WEIRD contexts, including settings which are economically and resource-constrained, participatory music is highly prevalent and utilized in daily life as well as in ritual and ceremonies. With due attention given to understanding the local context and culture in which these practices exist, there is considerable scope to co-develop culturally embedded interventions and approaches which are both culturally meaningful and aligned with research evidence. It is important to note that the ways in which music functions, in terms of its meaning, is highly specific to the individual context—there is no one size that fits all and local expertise will always be required to provide a nuanced understanding of the roles and affordances that participatory music can have. An approach which takes cultural practices as a starting point for the design of health interventions is only possible in the context of partnerships which span disciplines and involve broad representation at the community, civic and government level. This kind of interdisciplinary, partnership building approach can be challenging and requires negotiation of different cultural and disciplinary perspectives. Nevertheless, the co-design/co-creation approach is a way to facilitate this coming together of different perspectives. Concrete ways to bring about such cross-disciplinary working involve research councils co-funding projects where inputs across different disciplines are equally valued, where partners from LMIC and high-income country contexts are involved as equal members and where the time and resource allocation is appropriate to the extra demands such project entail. In general, the approach of measuring the health impact of initiatives or interventions that emerge from existing traditional cultural practices aligns well with broader recommendations [e.g. (Kola *et al.*, 2021)] to move to a strengths-based assessment of a community’s resources. This advocates for looking beyond the typical concept of resources (economic, access to biomedical resources) to consider the role that existing community groups, traditional healers, family structures and religious centres can all offer in terms of mitigating threats to physical and or mental health. 
